# Dogs’ insensitivity to scaffolding behaviour in an A-not-B task provides support for the theory of natural pedagogy

**DOI:** 10.1038/s41598-020-79557-8

**Published:** 2021-01-13

**Authors:** Patrick Neilands, Olivia Kingsley-Smith, Alex H. Taylor

**Affiliations:** 1 School of Psychology, University of Auckland, Auckland, 1010 New Zealand; 2 School of Biology, University of Auckland, Auckland, 1010 New Zealand

**Keywords:** Evolution, Animal behaviour, Psychology

## Abstract

Executive function plays a critical role in regulating behaviour. Behaviour which directs attention towards the correct solution leads to increased executive function performance in children, but it is unknown how other animals respond to such scaffolding behaviour. Dogs were presented with an A-not-B detour task. After learning to go through gap A to obtain the reward, the barrier was reversed, and the dogs had to inhibit their learned response and enter through gap B on the opposite side. Failure to do so is known as the perseveration error. In test trials, dogs taking part in one of two scaffolding conditions, a pointing condition, where the experimenter pointed to the new gap, and a demonstration condition, where the experimenter demonstrated the new route, were no less likely to commit the perseveration error than dogs in a control condition with no scaffolding behaviour. Dogs’ lack of responsiveness to scaffolding behaviour provides little support for suggestions that simple social learning mechanisms explains scaffolding behaviour in humans. Instead, our results suggest that the theory of natural pedagogy extends to the development of executive function in humans. This suggests that human children’s predisposition to interpret ostensive-communicative cues as informative may be an innate, species-specific adaptation.

## Introduction

Patiently waiting your turn, flexibly switching between two different tasks, and being able to learn a novel approach to solving an old problem; all these behaviours rely heavily on executive function, otherwise known as executive control. Executive functions are an important suite of cognitive mechanisms that are central to regulating behaviour and are crucial for problem-solving and task-completion^1^. Executive function takes many years to develop^2^ and there can be substantial individual differences in children’s performance in executive function tasks^3^. In humans, executive functions appear to be closely associated with the prefrontal cortex (PFC) ^4^. Similarly, the ability to flexibly regulate and control behaviour in other mammals appears to be mediated by the PFC^5–7^ and homologous regions of the brain in birds^8^, suggesting that executive function has deep evolutionary roots.

In humans there has been much interest in what factors may predict better executive control, as strong executive function ability correlates with stronger performance in school^9,10^, better health outcomes^11–13^, and reductions in anti-social behaviour^14^ amongst many other benefits^1^. While early research emphasized the importance of genetic influences on individual differences in executive function^15^, more recent research has recognized the impact that the socio-cultural environment can have on executive function development, particularly the social interactions between young children and adults such as their parents and teachers^16^. When interacting with children while they are completing a task or solving a problem, parents and teachers often exhibit a range of scaffolding behaviours. These behaviours, such as directing the child’s attention to salient parts of the task, encouraging them if they are on the right track, and redirecting their attention if they are on the wrong track, are a form of “autonomy support”^17,18^. Such behaviour enable children to better solve problems by themselves, and include both verbal and non-verbal actions^16^. For example, when young children have had experience of retrieving an object from location A several times and this object is subsequently removed from location A and placed at location B, children struggle to inhibit their learned response to search at location A, a mistake termed the ‘perseveration error’^19^. However, in A-not-B tasks where an experimenter points at location B before allowing the participant to search for the object, children are less likely to commit this error^20^. Such scaffolding appears to play a key role in the development of executive function: the extent to which parents engage in both verbal and non-verbal scaffolding behaviour predicts children’s degree of future executive control and their competency in skills such as maths and language^17,18,21–26^.

While, there is robust evidence that scaffolding improves both children’s executive function and learning outcomes, there is considerable debate regarding the mechanisms underpinning scaffolding behaviour^27^. Attentional accounts suggest that the main role of scaffolding behaviour is to help children overcome attentional inertia^28^ and focus on relevant parts of the task. In these accounts, successfully responding to scaffolding only requires a redirection of attention^20^ and thus even simple social learning mechanisms such as social facilitation or stimulus enhancement^29^ may be sufficient to respond successfully to scaffolding behaviour. In contrast, natural pedagogy theory argues that human infants are uniquely adapted to interpret ostensive-communication cues as informative from a young age^30–33^. That is, rather than ostensive-communication cues merely eliciting the infants’ attention, infants have an expectation that these cues will provide relevant and generalizable information^34,35^ that refers to a specific object or location^36–38^.

Dogs are an ideal species to test between the attentional accounts and the natural pedagogy accounts of scaffolding behaviour. Dogs are capable of learning socially via social facilitation^39^ and stimulus enhancement^40^, as well as more complex mechanisms such as imitation^41,42^ and, similarly to humans, appear to be sensitive to ostensive-communication cues^43,44^. However, despite both dogs and infants being sensitive to such cues, there appears to be stark contrasts in how they interpret these gestures. For infants, such gestures are declarative: a means with which to convey information about the world^45,46^. Correspondingly, infants use pointing gestures to both share^47^ and obtain^48^ information from an early age and prioritize ostensive-communication cues^49^ to the extent that they will reorganize their own knowledge on the assumption that such cues signal meaningful information^50^. In contrast, dogs appear to interpret ostensive-communicative cues as a means to either request^51^ or command^52,53^ but show little evidence of interpreting such cues as truly informative^52–55^. Instead, dogs appear to view such cues as being lightly imperative^52^: following these cues in novel contexts but tending to prioritize their own prior experience^56,57^. Therefore, if scaffolding functions by simply re-directing infant attention, we would predict that scaffolding behaviour should lead to a similar improvement in dogs’ performance in an inhibitory control task due to their sensitivity to human ostensive-communication cues. However, if infants’ response to scaffolding behaviour is built upon a uniquely human expectation that ostensive-communication cues are consistently and meaningfully informative, we would predict that scaffolding should not improve dogs’ performance in an inhibitory control task.

We tested how dogs respond to scaffolding behaviour by presenting them with an executive function problem: the A-not-B barrier task^58^, a test that has been widely used to test inhibitory control in humans and other animals^59^. In this task, dogs are presented with a barrier with a gap at one end and food on the other side. After having four trials to learn to go through the gap in order to obtain the food, dogs are presented with four test trials where the barrier is slid across so that the gap is now on the opposite side. In order to retrieve the reward, the dogs must inhibit their learnt response to approach the original gap, and instead approach the new gap. However, both dogs^58^ and children^60^ have a tendency to initially move towards the original gap, and so exhibit a perseveration error. In order to investigate whether scaffolding behaviour improves dogs’ performance in an executive function task, we had dogs take part in one of three conditions during the test trials. In the control condition, dog took part in four control trials where they received no scaffolding behaviour. In the pointing condition, dogs took part in four pointing trials where an experimenter dynamically pointed towards the new gap before the dog was released, and finally, in the demonstration condition, dogs took part in four demonstration trials where the experimenter walked through the new gap before the dog was released (see Supplementary video S1 for examples). Both pointing and demonstrating the route are forms of scaffolding behaviour as the experimenter uses ostensive-communication cues to gain the dogs’ attention before directing that attention towards salient parts of the task through their actions. If dogs are sensitive to scaffolding behaviour, we predict that dogs in the control condition would be substantially more likely to show the perseveration error than dogs in either the pointing or demonstration conditions.

## Results

**Figure 1:**
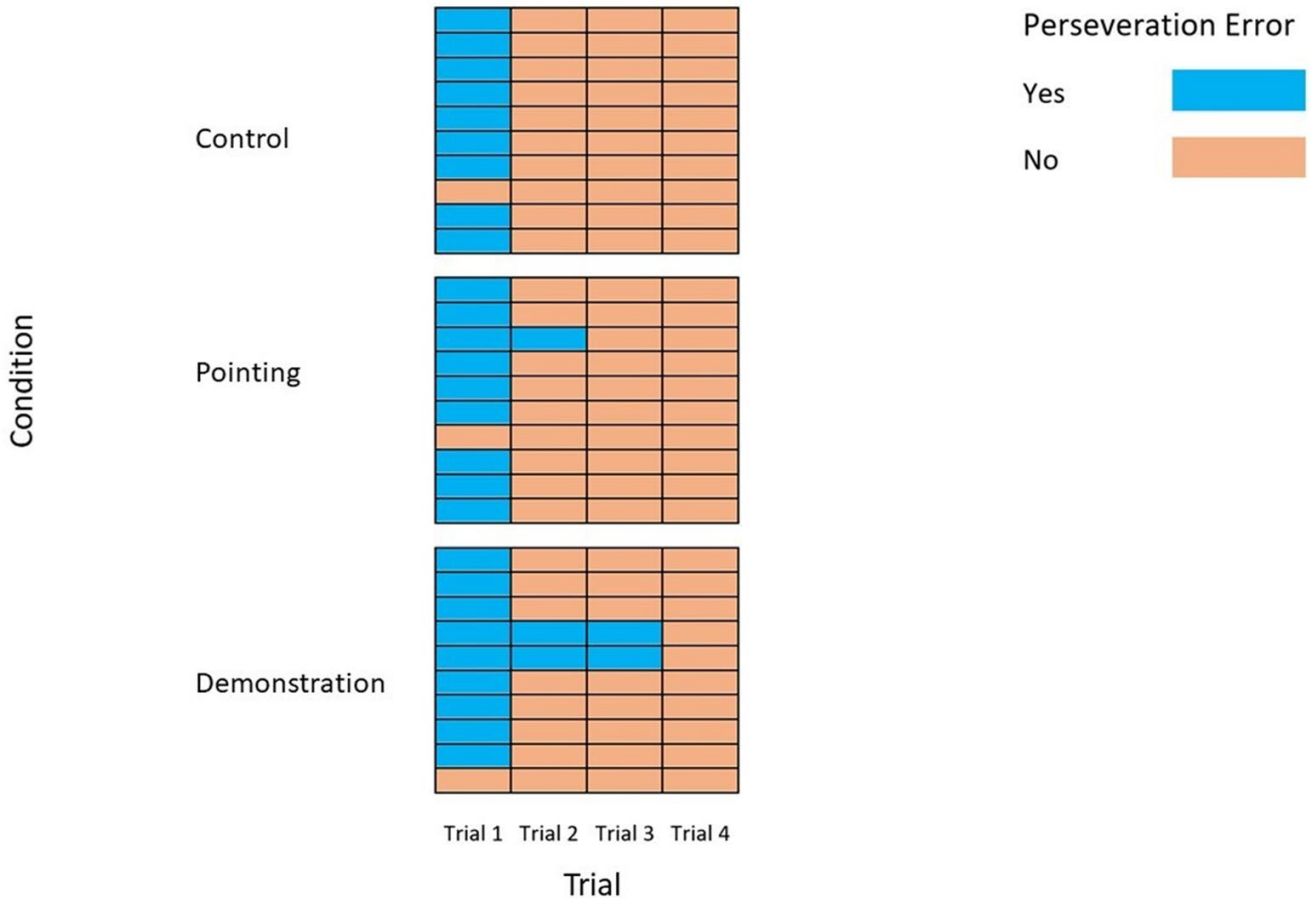
Dogs in all conditions were equally likely to commit the perseveration error on the first trial and as likely to learn to avoid committing it over all four trials. Each separate block represents a condition (with 10 dogs in each condition.) Each row represents an individual dog and each column represents an individual trial. 9/10 dogs in each condition showed the perseveration error on the first trial.

Dogs in all three conditions were substantially more likely than chance to commit the perseveration error on the first trial (Control Condition: 9/10 dogs, Bayesian Binomial test: BF = 18.5; Pointing Condition: 9/10 dogs, Bayesian Binomial test: BF = 18.5; Demonstration Condition: 9/10 dogs, Bayesian Binomial test: BF = 18.5; Fig. 1) and whether the experimenter engaged in scaffolding behaviour or not had no effect on how likely the dogs were to initially move toward gap A (Pointing vs Control Bayesian contingency test: BF = 0.329; Demonstration vs Control Bayesian contingency test: BF = 0.329).

Across all four trials (Fig. 1), the 10 dogs in each condition were substantially less likely than chance to commit the perseveration error (Control Condition: 11/40 trials, Bayesian Binomial Test: BF = 0.050; Pointing Condition: 10/40 trials, Bayesian Binomial Test: BF = 0.046; Demonstration Condition: 13/40 trials, Bayesian Binomial Test: BF = 0.061), suggesting that, over the course of the test trials, the dogs learnt to inhibit their initial response to move towards the original gap’s location. Dogs in either the Pointing (Pointing vs Control Bayesian contingency test: BF = 0.299) or Demonstration conditions (Demonstration vs Control Bayesian contingency test: BF = 0.176) were no less likely to commit the perseveration error than dogs in the Control condition. As such, the scaffolding behaviour directed towards the dogs appeared to have no effect on whether they initially showed the perseveration error on the first trial or how likely they were to learn to avoid making the error across all four trials.

Additionally, we ran exploratory analyses to examine if there were any differences between our conditions in how long dogs took to solve the task on their first trial, or if dogs differed in their route towards the barrier, which might indicate they were more hesitant in one condition than another. After controlling for how long dogs took to solve the task in the final learning trial, dogs’ latency to solve the task in the first test trial did not differ from the control condition (mean ± 95% CI: 4.59 ± 2.94 s) in either the pointing condition (2.32 ± 0.76 s; Bayesian independent t-test: BF = 0.812) or the demonstration condition (2.07 ± 0.51 s; Bayesian independent t-test: BF = 1.002). Similarly, in the first test trial, there was no difference in how long it took dogs to change direction after their initial movement towards the barrier in the control condition (2.37 ± 0.97 s) compared to either the pointing condition (1.96 ± 0.55 s; Bayesian independent t-test: BF = 0.480) or demonstration condition (1.67 ± 0.37 s; Bayesian independent t-test: BF = 0.710). Both these findings are consistent with the null hypothesis that scaffolding behaviour has no effect on dogs’ performance in an inhibition task.

## Discussion

Our results demonstrate that scaffolding behaviour such as pointing to a gap or demonstrating a route had no effect on how likely dogs were to commit the perseveration error in an A-not-B detour task. Furthermore, scaffolding behaviour did not improve dog’s efficiency at solving the task on the first test trial. These results cannot be explained in terms of a floor effect, where there is no difference between the conditions simply because the task was too difficult for the dogs to solve regardless of human intervention. Firstly, dogs clearly understood the contingencies of the task as, in the learning phase, all subjects were readily approaching the treat through the original gap by the end of this phase. Secondly, in the test trials, all dogs successfully inhibited the learned response to approach the original location of gap A by the final trial. Similarly, a ceiling effect, where there was no difference between conditions because the task was too easy for the dogs, also cannot account for this result. Across all three conditions, dogs were substantially more likely than chance to commit the perseveration error on the first trial, suggesting that inhibiting the initial movement towards the original gap was challenging for the dogs. Furthermore, dogs’ failure to use the actions of the experimenter to improve performance in the pointing and demonstration conditions does not appear to be attributable to a lack of sensitivity to these actions. Past research has shown that dogs are highly sensitive to pointing gestures from humans^61–63^, including when humans point towards locations rather than objects^56^, and are able to learn from the routes taken by humans to solve detour tasks^64,65^. Therefore, our results strongly suggest that, despite sensitivity to referential communication from humans^43,44^, dogs do not respond to scaffolding behaviour by showing better inhibitory control in an A-not-B detour task.

Dog’s lack of sensitivity to an experimenter’s scaffolding behaviour in this current study contrasts sharply with the improvement in children’s performance in an A-not-B task after an experimenter points at the correct location^20^. This difference in responsiveness may appear surprising, considering that simple social learning mechanisms such as social facilitation or stimulus enhancement seem to be sufficient to explain the effectiveness of scaffolding behaviour. However, our results suggest that responding to scaffolding takes more than simple social learning mechanisms and a sensitivity to ostensive-communicative cues. In humans, this scaffolding behaviour is embedded within a context where infants have an expectation that these cues will provide relevant and generalizable information^34,35^ that refers to a specific object or location^36–38^. Without that expectation^51^, it seems dogs’ personal experience of having previously completed the task in the learning trials resulted in the dogs regarding the experimenter’s ostensive-communicative gestures as not relevant, resulting in the dogs not attending to the salient part of the task and thus being less able to inhibit their learned response to approach the original location of the gap.

In sum, while it has been suggested that children’s attentiveness to ostensive-communication cues is merely the by-product of general social learning mechanisms and children’s particular socio-cultural environment^66–68^, our results support the claim made by natural pedagogy theory: children’s responsiveness to ostensive-communication cues is an innate, species-specific adaptation^30,31^. Furthermore, our findings suggest that the natural pedagogy adaptations of humans appear to not only help with the learning of complex information but also play a role in the development of executive function in young children.

## Methods

### Ethics statement

The present study was approved by the University of Auckland Animal Ethics Committee R001826 and the University of Auckland Human Ethics Committee R018410. All work with the dogs was in accordance with the guidelines of the New Zealand National Animals Ethics Advisory Committee. Dogs were recruited through owners’ responses to online applications. Written informed consent for participating in this study was obtained from the owners.

### Participants

A total of 30 dogs were recruited, with ten dogs in each condition. To determine sample size, we used a Bayesian stopping rule where we collected data until we had at least 10 dogs in each condition and Bayes Factors of > 3 or < 0.333 in our contingency tests. All dogs were pet dogs (aged 2–10 years old) which were accompanied to the lab by their owners (see Supplementary Dataset S1 for details for dogs included in the study).

### Experimental set-up

**Figure 2:**
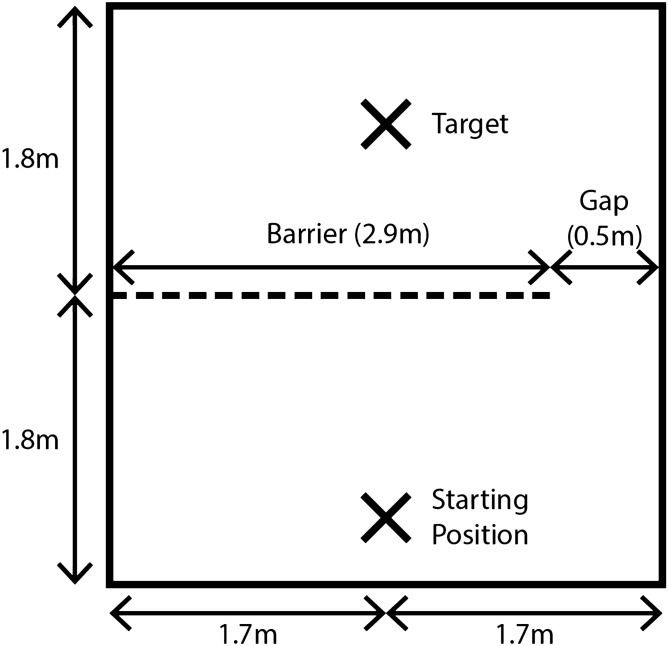
Set up of experimental room. The subject was placed on the starting position opposite the experimenter. In the learning trials, the experimenter would get the dog’s attention, show it the treat, place the treat in a bowl, and place the bowl on the target. After the experimenter gave the release command, the owner would release the dog and the dog would have to enter through the gap in the barrier to reach the treat. The test trials were the same as the learning trials, but the gap was moved to the other side. The experimental procedure was the same except that in the scaffolding conditions, the experimenter would either point to the gap or demonstrate the route before giving the release command.

The experiment took place in a 3.6 m × 3.4 m testing room. A 2.9 m barrier was placed across the middle of the room, with a 0.5 m gap between either the left or right end of the barrier (gap A) and the wall. Which side the original gap was on was counterbalanced across dogs (see Fig. 2 for set up of testing room).

### Protocol

Dogs took part in four initial learning trials. Using the same approach as previous research^58^, the experimenter entered the room first and positioned themselves in the middle of the room, directly behind the barrier. The dog was then positioned on a mark opposite the experimenter, 1.6 m away from the barrier. After the dog was settled and facing the experimenter, the experimenter would show the dog a treat before placing it in a bowl and putting the bowl on the ground on their side of the barrier. Once the bowl was placed on the ground, the experimenter called the dog’s name and gave the release command “ok, go!”. In order to avoid inadvertently cuing the dog, both the owner and experimenter were asked to stare straight ahead and avoid looking towards the gap. Additionally, both the owner and experimenter were blind to hypotheses. After the dog was released, the trial ended after the dog obtained the reward. Dogs received four learning trials before moving onto the test trials.

Dogs took part in four test trials in one of three conditions. In all conditions, the barrier would be slid across the room so that the gap was now at the opposite end of the barrier (gap B). In all conditions, the dog would be brought back into the testing room and positioned on the mark as before. In the Control condition, the test trials were carried out in the same manner as the learning trials. In the pointing condition, the experimenter would bait the bowl as before but after placing the bowl on the ground, the experimenter would call the dog’s name, look it in the eyes, point to the new gap, and then call the dog’s name again whilst alternating their gaze between the gap and the dog. After calling the dog’s name, the experimenter gave the release cue as in the learning trials and maintained the pointing gesture until the dog had retrieved the reward. In the Demonstration condition, the dog would enter the room and be placed on the mark as before, but the experimenter would be standing, opposite gap B, on the same side of the barrier as the dog. Once the dog was settled, the experimenter would show the dog the treat, call its name and make eye contact with dog twice. Once the dog was attending to the experimenter, the experimenter would walk through the new gap to their original position facing the dog. Afterwards, the experimenter would place the food down and give the release cue as in the leaving trials.

### Analysis

The initial movement that the dogs made was coded (either towards the original location of gap A or the new gap B). Bayesian binomial tests were used to determine whether dogs were more likely than chance to commit the perseveration error and move towards the original location of gap A in each condition, and Bayesian contingency tests were used to test whether the number of dogs committing the perservation error differed between the Control condition and the two scaffolding conditions. Bayes Factors > 3 indicate substantial support for the alternative hypothesis, while Bayes Factors < 0.333 indicate substantial support for the null hypothesis. As dogs tend to show a learning effect over the course of detour trials^69^, we primarily focused on dogs’ performance in the first trial for our analyses. The Bayesian binomials had Beta priors of 1 for both parameter a and parameter b. The Bayesian contingency test was an independent multinomial with rows fixed and had a prior concentration of 1.

Alongside our confirmatory analyses, we also carried out exploratory analyses looking at whether the experimenters’ scaffolding behaviour improved dog’s efficiency at solving the task, regardless of whether they showed the perseveration error or not. As the majority of dogs learned to avoid committing the perseveration error by the second test trial, we focused on the first test trial for these analyses. Firstly, we compared the how quickly it took dogs to solve the first test trial across condition. In order to control for individual differences in motivation, we divided the latency for dogs to complete the first test trial by the latency of dogs to complete the final learning trial. Secondly, we compared how long dogs persisted in moving in the initial direction they chose towards the barrier before changing the direction. Both comparisons were made using Bayesian independent t-tests. The priors for both t-tests were Cauchy distributions centred around a mean effect size of zero. The full, rather than directional Cauchy distribution was used to reflect the exploratory nature of these analyses.

All analyses were carried out in Jasp 0.10 (Jasp Team, 2019).

## Supplementary Information

Supplementary Information.

Supplementary Video 1.

## Data Availability

All data generated and used for analysis in this study are found in Supplementary Dataset S1 in the Supplementary Information Files.
